# Cooperative excitonic quantum ensemble in perovskite-assembly superlattice microcavities

**DOI:** 10.1038/s41467-019-14078-1

**Published:** 2020-01-16

**Authors:** Chun Zhou, Yichi Zhong, Hongxing Dong, Weihao Zheng, Jiqing Tan, Qi Jie, Anlian Pan, Long Zhang, Wei Xie

**Affiliations:** 1 Key Laboratory of Materials for High-Power Laser, Shanghai Institute of Optics and Fine Mechanics, Chinese Academy of Sciences, 201800 Shanghai, China; 2 Center of Materials Science and Optoelectronics Engineering, University of Chinese Academy of Sciences, 100049 Beijing, China; 3 State Key Laboratory of Precision Spectroscopy, Quantum Institute for Light and Atoms, School of Physics and Electronic Science, East China Normal University, 200241 Shanghai, China; 4 Hangzhou Institute for Advanced Study, UCAS, 310024 Hangzhou, China; 5 Key Laboratory for Micro-Nano Physics and Technology of Hunan Province, College of Materials Science and Engineering, Hunan University, 410082 Changsha, China

**Keywords:** Nanocavities, Polaritons, Quantum dots

## Abstract

Perovskites—compounds with the CaTiO_3_-type crystal structure—show outstanding performance in photovoltaics and multiparameter optical emitters due to their large oscillator strength, strong solar absorption, and excellent charge-transport properties. However, the ability to realize and control many-body quantum states in perovskites, which would extend their application from classical optoelectronic materials to ultrafast quantum operation, remains an open research topic. Here, we generate a cooperative quantum state of excitons in a quantum dot ensemble based on a lead halide perovskite, and we control the ultrafast radiation of excitonic quantum ensembles by introducing optical microcavites. The stimulated radiation of excitonic quantum ensemble in a superlattice microcavity is demonstrated to not be limited by the classical population-inversion condition, leading to a picosecond radiative duration time to dissipate all of the in-phase dipoles. Such a perovskite-assembly superlattice microcavity with a tunable radiation rate promises potential applications in ultrafast, photoelectric-compatible quantum processors.

## Abstract (web summary)

The realization and control many-body quantum states in perovskites would extend their application to ultrafast quantum operation. Here, the authors generate a cooperative quantum state of excitons in a lead halide perovskite quantum dot ensemble and control the ultrafast radiation through optical microcavites.

## Introduction

Perovskites are an excellent optical candidate for achieving large oscillator strength and highly efficient light absorption^1–4^. However, realizing long-range quantum states based on perovskite systems and controlling their quantum behavior to provide a fast response and high sensitivity are significant challenges. To overcome these issues, the quantum features of materials can be revealed by reducing their dimensions^5^. For example, one can uncover the quantum character of perovskites by using perovskite quantum dots (QDs). In addition, long-range phenomena involve many-body cooperation^6–9^. To combine these two kinds of performance to develop long-range quantum behavior, a QD-assembly superlattice^10,11^ is an excellent candidate. The QD-assembly superlattice is a two- or three-dimensional structure in which same-size QDs are the basic unit, and they arrange themselves periodically. Closely packed semiconductor QD superlattices with long-range order could offer a high density of exciton states, low energy broadening, and long dephasing time of particles, all of which enable the formation of macroscopic quantum states^12^. On the other hand, in order to control these collective behaviors for applications, further requirements arise, such as new degrees of freedom for manipulation. Although various structures and devices based on the perovskite family have been designed for highly efficient solar cells^1,2^, highly coherent single-photon source^13^, and other light sources with excellent properties^14–16^, an ultrafast control of many-body quantum ensemble in a perovskite core has never been reported.

**Fig. 1:**
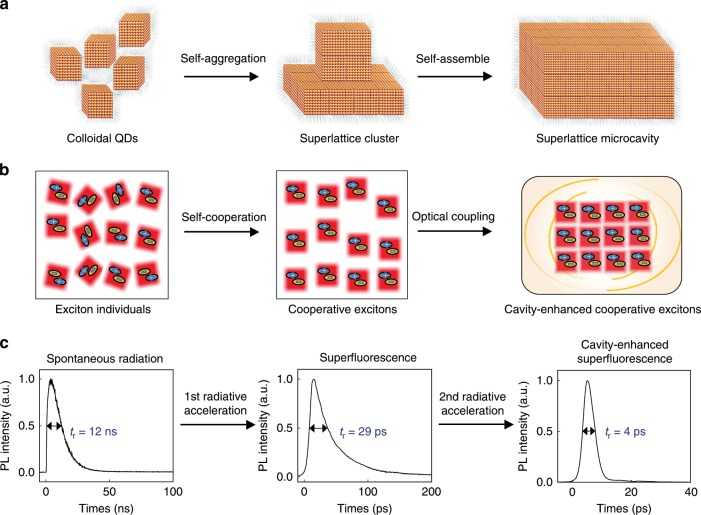
Linking “self-assembly of QDs” to “phase transitions of exciton ensemble”, and “optimization of radiative features”. **a** Self-assembly evolution from monodisperse colloidal QDs (left) to a closely packed SC (middle) and finally to a highly symmetric QDSM (right). The whiskers around the cubic QD are oleylamine and oleic acid ligands. **b** Phase transitions of exciton ensemble in the structures discussed above. The “+” and “−” orientations indicate the phase of dipole moments. **c** Experimental radiation dynamics. Monodisperse QDs radiate spontaneously with a radiative time *t*_r_ (the full-width at half-maximum (FWHM) of the dynamical PL peak) of tens of nanoseconds, while self-cooperating excitons in the SC emit a SF pulse with *t*_r_ on the order of 10 ps. Furthermore, the cavity field in the QDSM accelerates the SF process, with *t*_r_ reaching as low as a few picoseconds.

**Fig. 2:**
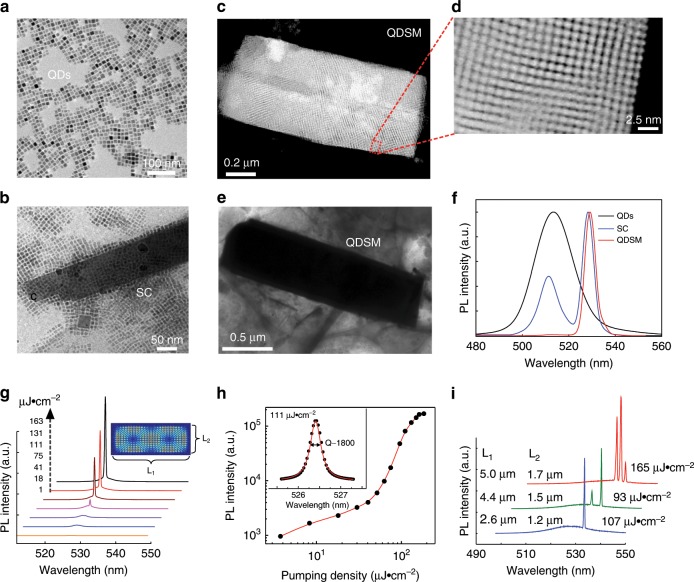
Dynamic tracing and optical characterization of perovskite QDSMs. **a** TEM image of monodisperse CsPbBr_3_ cubic QDs. **b**–**e** TEM images of the evolution of monodisperse QDs (**a**) self-assembling into closely packed SC (**b**) and finally to the rectangular QDSM (**e**) via low-temperature aging and drying-mediated methods. Rows of dots capped with oleylamine and oleic acid arrange themselves along the crystalline axis (**b**) and finally form sharp edges and regular shape (**e**). **c** is the annular TEM images of the cuboid QDSM and **d** is a close-up view of the red dotted box in **c**. **f** PL spectra of different structures. **g** PL spectra of the cavity-enhanced SF effect from a QDSM at 77 K. Inset: the simulated field distribution of the whispering gallery mode, L_1_ and L_2_ are long side and short side of the QDSM, respectively. **h** Power dependence of the PL intensity in cavity mode. Inset: Lorentz fitting of the cavity mode. **i** The resonant optical modes of QDSMs with different sizes. Multimode lasing can be obtained and the spacing between two adjacent modes decreases with the increasing of cavity size.

**Fig. 3:**
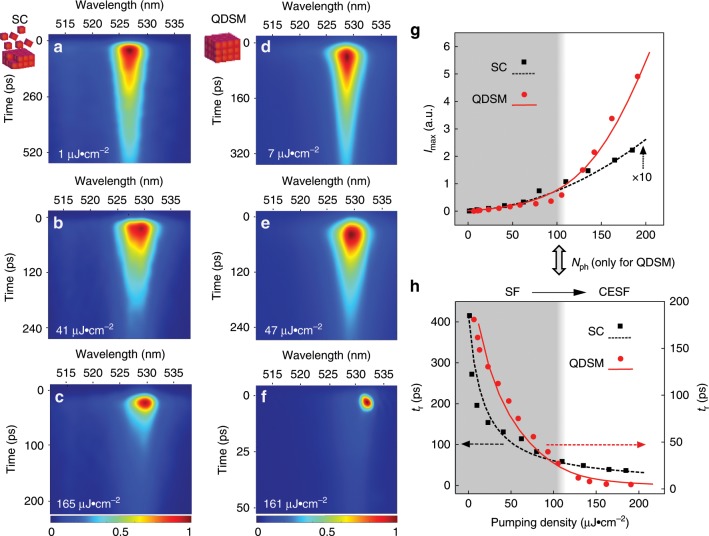
Dynamics of SF and CESF under different pumping densities. **a**–**c** Streak-camera images of SF behavior in a SC sample at 77 K. **d**–**f** Corresponding data for CESF in a QDSM sample. **g** Peak intensity *I*_max_ of SF/CESF signals vs. pumping density. The dots represent experimental data and the lines represent theoretical simulations. **h** Power dependences of radiative time *t*_r_. As shown in **g**, **h**, the CESF effect in the QDSM exhibits an obvious pumping threshold. Here, *N*_ph_ represents the threshold density of stimulated photon amplification in the QDSM.

**Fig. 4:**
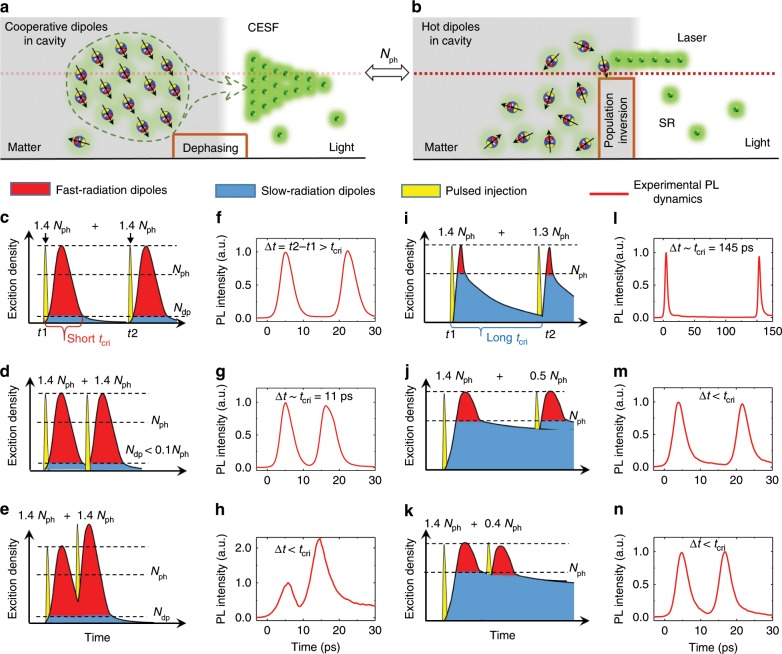
Surplus dipoles after the CESF process vs. the case of a classical laser. **a**, **b** Sketches of cooperative (hot) dipole ensembles in optical cavities. SR means spontaneous radiation. **c**–**e**, **i**–**k** Dynamics of exciton density in a cooperative (hot) dipole system. The classical lasing process ends at the density level *N*_ph_, and CESF is limited by the small dephasing density *N*_dp_. **f**–**h**, **l**–**n** Experimental PL dynamics in the corresponding system. The PL intensity is normalized by that of the first pulse. The critical time *t*_cri_ of cooperative quantum excitons is much shorter than that of a hot exciton ensemble. The threshold density *N*_ph_ for the QDSM sample and that for the classical lasing sample is about 70 and 50 μJ cm^−2^, respectively.

Here, we develop a novel microstructure—a “QD superlattice microcavity (QDSM)”—for controlling many-body quantum behaviors with high response rate (Fig. 1). In light-matter interaction system, two major types of methods can manipulate the many-body effect and accelerate the coupling rate. One strategy involves the material itself, for example, transforming the many-body system into a collective state wherein the cooperative ensemble behaves like a giant quasi-particle with large oscillator strength^17–21^. The other method is related to the optical environment coupling. For example, optical cavities can be constructed to stimulate the material source to radiate coherently^22–24^. Our QDSM design combines the advantages of these methods. More specifically, we integrate the high quality of a QD superlattice and the optical controllability of a cavity in a perovskite microstructure (Fig. 2 and Supplementary Fig. 2). Such a highly symmetric, long-range-ordered perovskite QDSM could show both superfluorescence (SF) behavior and an optically stimulated amplification effect (i.e., lasing) above the critical excitation density. The entire radiation process, which we term cavity-enhanced SF (CESF), is realized at 77 K with high repeatability (Fig. 3). In addition, cooperative excitons exhibit quantum behavior during their lasing process, in which the perovskite system consumes all the cooperative components of dipoles by the CESF channel, rather than being limited by the population-inversion condition, as in classical lasers (Fig. 4). By utilizing such a unique characteristic, the radiation time of cooperative exciton ensemble is shortened to be picoseconds.

## Results

### Structural characterizations

High-quality QDSMs are obtained using an innovative self-assembly method. First, monodispersed CsPbBr_3_ QDs (Fig. 2a, Supplementary Fig. 1) are synthesized according to a previously reported method with modifications^25^. Transmission electron microscopy (TEM) and fast Fourier transform reveal that the CsPbBr_3_ QDs are cubic with very good crystallinity (Supplementary Fig. 2). These cubes self-assemble into regular geometric configurations seamlessly and tightly on the silicon wafer. Their size can be controlled (from hundreds of nanometers to dozens of micrometers) by varying the aging time at low temperature and the evaporation rate of the solution (Fig. 2b, e). During this process, low-temperature operation decreases the thermal energy of the whole system so that the initially repulsive QDs become attractive^26^. Next, slowly evaporating the solution in vacuum enables QDs of the same size to aggregate and align perfectly^27,28^, thus achieving long-range ordering and yielding regular geometrical superstructures (Fig. 2c, d). This assembly method produces samples with few defects and low inhomogeneous broadening, which facilitates the cooperation of excitons. Moreover, these samples remain stable in the ambient atmospheric environment for several months and even maintain good fluorescence after high-power laser pumping. These excellent properties demonstrate the good performance of our innovative self-assembly method, which may be extended to the self-assembly of other semiconductor nanoparticles.

### Optical properties of QDSM

The dynamic process of QD self-assembly can be investigated via photoluminescence (PL) spectroscopy. The PL spectrum of a superlattice cluster (SC) is compared with that of monodispersed QDs in Fig. 2f, wherein a new peak emerges for the SC at around 528 nm, which originates from the overlapping of single-QD excitonic wave functions in the superlattice structures^10^. In addition, QDs of the same size have superiority in the self-assembly process, thus narrowing the original broad peak. More interestingly, during the increase of the degree of self-assembly, the original peak of the unassembled QDs disappear, and the new peak dominates the PL spectrum of the QDSM. Furthermore, CESF behavior is observed in the optical modes of QDSM (Fig. 2g). The energy centers of the optical modes and the mode interval can both be controlled by adjusting the size of the QDSMs (Fig. 2i). Whispering gallery modes are formed by total reflections, and the field distributions of the resonant modes in the microcavities fit well with the theoretical prediction of whispering gallery model (Supplementary Fig. 3). The typical *Q* factor of QDSM can reach about 2000 (Fig. 2h), corresponding to an energy broadening of cavity mode of ~1 meV and a photon lifetime on the order of 1 ps. Generally, our sample is <1 µm thick, and its lateral dimensions are several micrometers. To confine light for 1 ps in such a small cavity, the light must reflect at the external surface of QDSMs tens of times, using a simplified classical picture. Thus, to maintain the high quality of the microcavity assembled from QDs, it must possess smooth external surfaces and few internal defects.

### Dynamics of SF and CESF

The typical SF/CESF characteristics of a SC/QDSM are presented in Fig. 3 based on time-resolved PL (TRPL) measurements under different excitation densities. The radiative time (*t*_r_), that is, the full-width at half-maximum of the dynamical PL peak, decreases rapidly with increasing pumping density. Meanwhile, the dynamical PL peak intensity (*I*_max_) increases non-linearly during the corresponding process. Both are fitted well by the results of a theoretical simulation (Supplementary Note 2 and Supplementary Fig. 7). Notably, the *I*_max_ of CESF exhibits an obvious intensity threshold (*N*_ph_), which represents the critical density of stimulated photon amplification in the QDSM. Moreover, above this threshold, the CESF effect could attain a much smaller *t*_r_ than that of SF due to the radiative enhancement by coupling with the amplified light field. These characteristics of CESF are common for different QDSM samples. The value of *N*_ph_ is related to the quality of the sample and takes values ranging from tens to hundreds of microjoules per square centimeter per pulse. In addition, compared with the radiation time of individual QDs (~12 ns) (Fig. 1c, Supplementary Fig. 4), *t*_r_ of SF is about 400 times shorter (~30 ps), while *t*_r_ of CESF is 3000 times shorter (~4 ps). The reduced ratios *t*_SR_/*t*_SF_ roughly indicates the effective numbers of cooperative dipoles *N*_eff_ ~10^2^.

### Cooperative ensemble breaks population-inversion limitation

The nonlinear and dynamical characteristics of the CESF effect in cavities are easily confused with the normal lasing effect in semiconductor cavities. For example, the light signals that they emit have some similar features. Nevertheless, they are essentially different from the aspect of matter^29^, that is, the emitting core. Dense excitons in perovskite QDSMs are in a collective quantum state rather than being an ordinary thermal gas, as they are in a classical semiconductor laser system. For a cooperative dipole ensemble, a CESF pulse will rapidly dissipate all of the in-phase dipoles, while the dephasing component of the dipoles would radiate non-collectively and slowly^8^. The residual density after CESF is determined by the dephasing dipoles in ensemble *N*_dp_ (Fig. 4a, c). In our samples, the *N*_ph_/*N*_dp_ ratio could be 10:1 or higher for the dipoles resonant with the cavity mode, and the fluctuation in the pulse energy of the pumping laser is about 5%. We focus on the unambiguous part of the PL data, wherein the intensity is larger than the fluctuation noise. The surplus ratio after the CESF process is close to this noise limit. However, the stimulated radiation of hot excitons in semiconductor lasers depends on the population-inversion condition^30^ (Fig. 4b, i). When the lasing process finishes, the semiconductor laser system remains a dense exciton ensemble, the density of which is limited by the threshold *N*_ph_. Experimentally, two pumping pulses with different time intervals and power densities (Supplementary Fig. 5) are used to test the residual excitons after the fast-radiation process (Fig. 4f–h, l–n). A conventional microlaser of the same perovskite material (i.e., a microspherical CsPbBr_3_ bulk crystal^31^) is selected to carry out a comparative experiment. The *Q* factor of the microsphere and the lasing threshold *N*_ph_ are similar to those of the QDSM sample (Supplementary Fig. 6). However, the microsphere shows a classical carrier response, which is totally different from that of QDSM sample. Thus, we experimentally demonstrate that the cavity-mediated cooperative quantum state could break the limitation of the population-inversion condition in the radiation process.

## Discussion

**Fig. 5:**
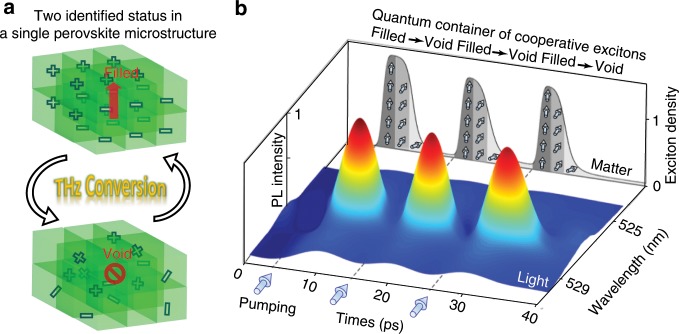
Terahertz conversion based on a CsPbBr_3_ QDSM. **a** The schematic illustrations of the “Filled“ and “Void” states for the perovskite quantum container. **b** Experimental demonstration of a quantum container with an ultrafast conversion of excitonic quantum ensemble, accompanying with the optical response of the CESF signals. The blue arrows along the *x*-axis indicate the excitation times. The arrows along the side plane represent the population status of the many-body quantum system.

Based on such a quantum feature of cooperative excitons, we propose a single perovskite QDSM as a THz quantum container, as shown in Fig. 5a. Here, we focus on rapidly manipulating the emitting core rather than the light field. We could define two different states for the perovskite QDSM as the two identified states of a quantum container. The existence of an excitonic quantum ensemble represents the “Filled” status, whereas the absence of cooperative dipoles indicates the “Void” status. The container can be “filled” via optical pumping. The excitation laser will rapidly yield dense carriers^30^ (<1 ps), but it is not responsible for setting up a cooperative quantum state, which is actually supported by the high-quality QDSM and the low-temperature environment placed the dense exciton ensemble. The formation time of cooperative states is short (~ps) because the cavity excitons with a small dipole distance can cooperate with each other via the efficient exchange of virtual photons^12^. However, the emptying process is a shortcoming for the fast control, wherein the excitonic quantum state is disappearing. Here, we use the radiative relaxation channel of CESF to empty the quantum container. Note that after the ultrafast CESF process, the residual carriers are dephased, and the quantum QDSM container is considered to be “Void.” Thus, the quantum container can be directly and rapidly controlled between the “Filled” and “Void” states. The lower limit of the control period (*t*_cri_, shown in Fig. 4c) could reach as low as 10 ps.

In summary, we propose the ultrafast control of a perovskite core of non-equilibrium cooperative excitons. We experimentally demonstrate it based on a closely packed perovskite QDSM. Such a many-body quantum device is not limited by the classical population-inversion condition and shows a unique radiation capacity with a tunable bandwidth of up to 0.1 THz. In addition to the collective manipulation in a perovskite system, we develop a novel microstructure, a “QDSM,” which can find application for various materials in designing micro-devices with long-range quantum features and high optical controllability.

## Methods

### Preparation of Cs-oleate

The preparation of Cs-oleate proceeded by the following method. CsCO_3_ (0.825 g, Aladdin, 99.9%), octadecene (40 ml, Sigma-Aldrich, 90%), and oleic acid (2.5 ml, Aladdin, >90%) were mixed in a 100 ml three-neck flask and dried under N_2_ for 1 h at 120 °C, and then heated under N_2_ to 150 °C until all the CsCO_3_ reacted with OA. The resulting Cs-oleate were stored for the next experiment.

### Synthesis of CsPbBr_3_ QDs

The synthesis of CsPbBr_3_ QDs is according to the previously reported hot-injection method with minor modifications^25^. Octadecene (4 ml, Sigma-Aldrich, 90%) and PbBr_2_ (69 mg, Aladdin, 99.999%) were mixed in a 50 ml four-neck flask and dried under N_2_ for 50 min at 120 °C. Then, oleylamine (1 ml, Aladdin, 80–90%) and oleic acid (0.5 ml, Aladdin, >90%) were injected into the flask; after 20 min at 120 °C under N_2_, the temperature was raised to 170–190 °C (for tuning the nanocrystals size), hot Cs-oleate (0.4 ml, 0.1 M in ODE, prepared above) was rapidly injected, and 5 s later, the reaction mixture was cooled in the ice-water bath. The resulting NCs were dispersed into toluene for self-assembly.

### Self-assembly of CsPbBr_3_ QDs into superlattices

The CsPbBr_3_ QD superlattice is formed in the solvent by low temperature aging and slow evaporation of solvent (toluene). Various superlattices with different sizes were made by tuning the low-temperature aging (10 °C) time and solvent evaporation rate. The aging time is about 4–15 days and the evaporation rate should be slower by putting the QD solution in vacuum.

### Characterization techniques

TEM, high-resolution TEM, and high angle annular dark field-TEM measurement were performed on a Tecnai G2 F20 S-TWIN operated at 200 kV. All the samples are previously dropped on clean bare wafer with fine concentration and later transfer onto 300-mesh copper TEM grid by spot cleaning.

### PL spectra and dynamical measurements

The PL spectra in Fig. 2 were measured under 400 nm femtosecond (fs) laser (Libra, Coherent, ∼40 fs, 10 kHz) with a confocal microphotoluminescence system (LabRAM HR Evolution). The system was equipped with a cryostat (80–475 K, Janis ST-500) and temperature controller (cryocon 22C). Liquid N_2_ was used for cooling. All the optical measurements were performed at 77 K. The dynamical measurements were performed on a streak camera (C10910–05, M10911–01) with a closed-cycle high-vacuum dewar (MONTANA) at 77k. The excitation source is fs pulsed laser (Spectra-Physics, ~400 nm, 80 MHz). It was divided into two or three beams of controllable power ratio and time intervals for the experiments in Figs. 4 and  5. The radiative efficiency would change with the excitation configure (such as the heating effect in the two or three beam case). Thus, the intensity-normalized data were used to indicate the density of surplus dipoles. Radiation dynamics were performed on a streak camera (C10910–05, M10911–01). We measure the spectral and temporal properties of luminescence in the reflection geometry. Luminescence is collected through the objective, separated from the reflected specular and scattered pumping laser light with a notch filter, and then directed to a spectrometer with CCD or the streak camera. The corresponding spectral and temporal resolutions are ~0.1 nm and ~1 ps, respectively. For all the experiments, the front surface of the sample is positioned at the focal plane of a high-numerical-aperture microscopy objective (NA = 0.42, ×50). A monochromated light (375 nm) from a xenon lamp equipped with a Horiba FluoroLog-3 spectrofluorometer in reflection geometry is used to perform TRPL measurements of Fig. 1c and Supplementary Fig. 3a. The emission is passed through a 500 nm blaze grating monochromator (iHR320) and collected by a TCSPC detector.

## Supplementary information

Supplementary Information

## Data Availability

All data in the manuscript and the Supplementary Materials are available from the corresponding author upon reasonable request. The source data underlying Figs. 1c, 2f, g, h, i, 3g, h, 4f, g, h, l, m, n and Supplementary Figs. 3a–c, 4a–c, 6b–d, 7a–d are provided as a Source Data file.

## Source data

Source Data
