# Supplementary material for: Cooperative excitonic quantum ensemble in perovskite-assembly superlattice microcavities
Source: Nat Commun. 2020 Jan 16;11:329. doi: 10.1038/s41467-019-14078-1 (PMC6965136; doi:10.1038/s41467-019-14078-1)
Supplement: Supplementary file 1 — Supplementary Information [file 41467_2019_14078_MOESM1_ESM.docx]

**Cooperative excitonic quantum ensemble in perovskite-assembly superlattice microcavities**

Zhou et al.

**Supplementary Note 1: Extended Experimental Data**


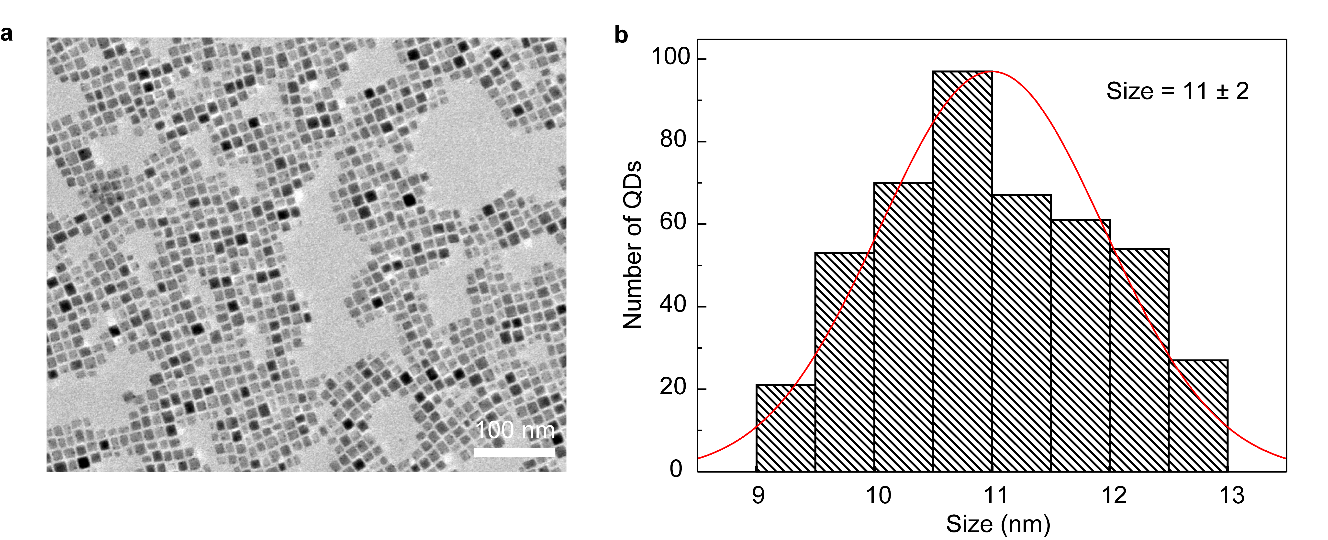


**Supplementary Figure 1. Size distribution and statistics of QDs used for self-assembly. a**, Low resolution TEM of monodispersed QDs. **b**, Histogram of QDs size distribution from Supplementary Figure 1a (QDs number > 500). The average size is 11 nm, with deviation of ±2 nm.


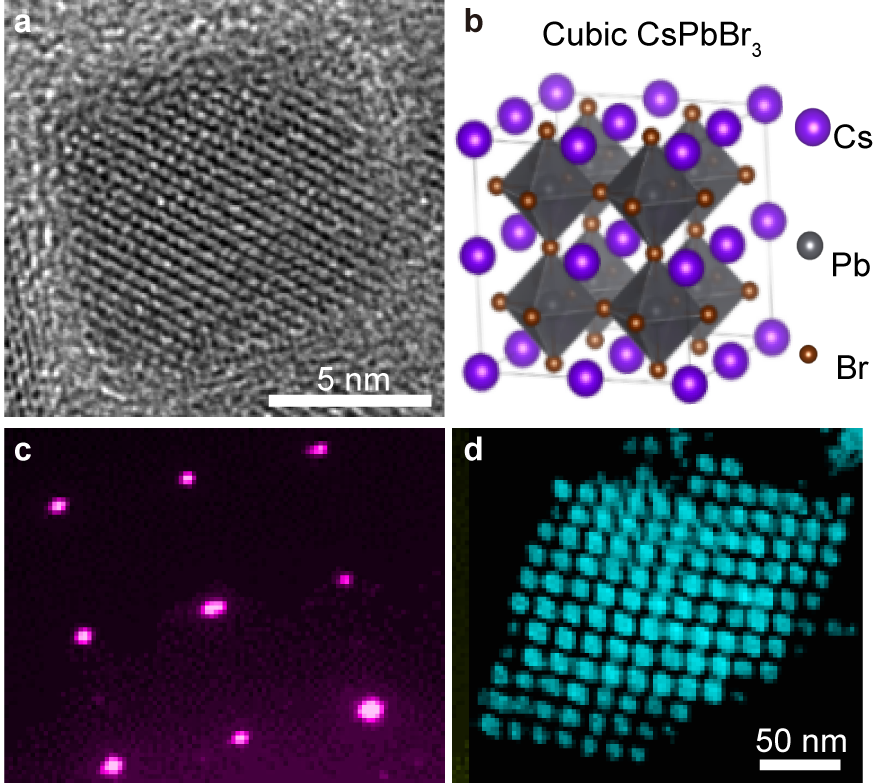


**Supplementary Figure 2. Characterization of single CsPbBr_3_ QD and monolayer QD superlattice.** **a-c,** High-resolution TEM image of a single CsPbBr_3_ QD (a), the corresponding Fourier transform (c), and the cubic lattice (b). **d,** High-angle annular dark-field imaging-TEM images of a monolayer QD superlattice


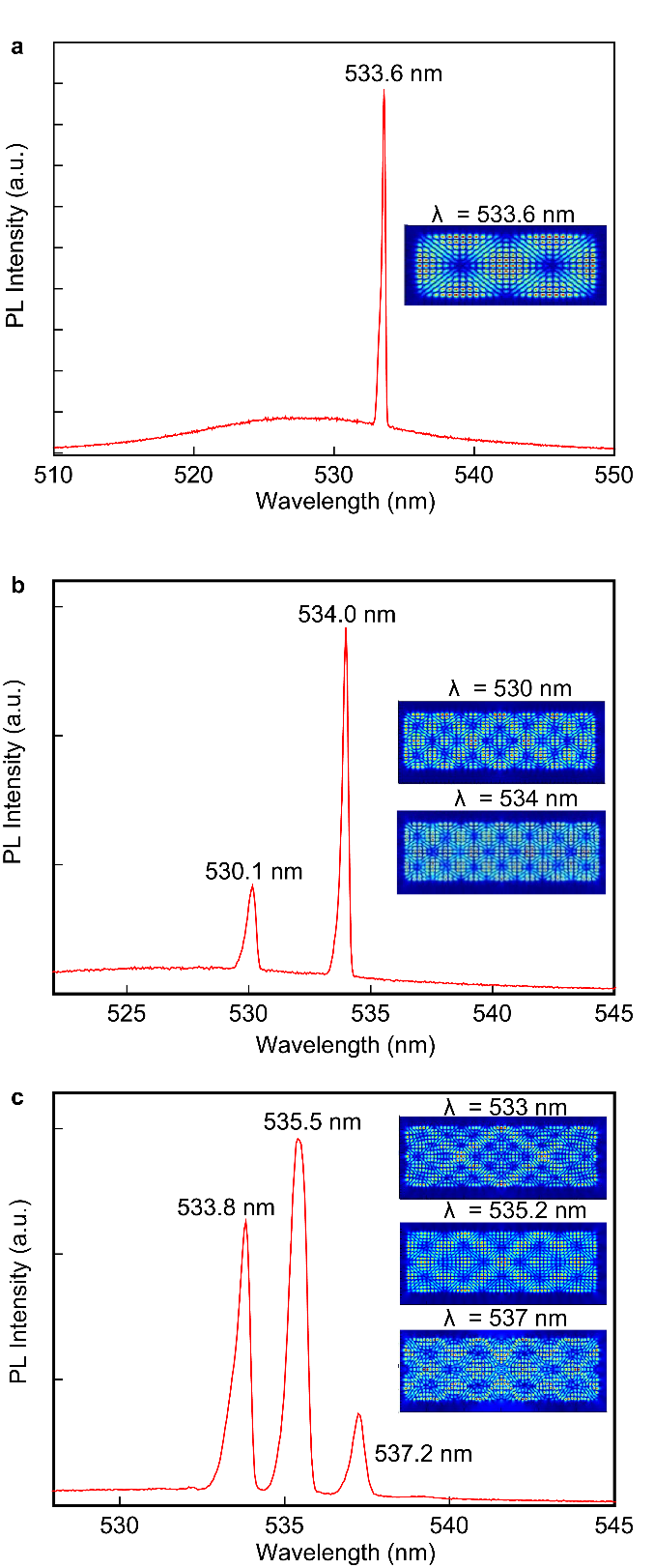


**Supplementary Figure 3. The lasing spectra of three typical QDSMs with different sizes.** Insets: the theoretical field distribution of the resonant lasing modes by FDTD simulation. Standing waves are confined in the microplate optical cavity.


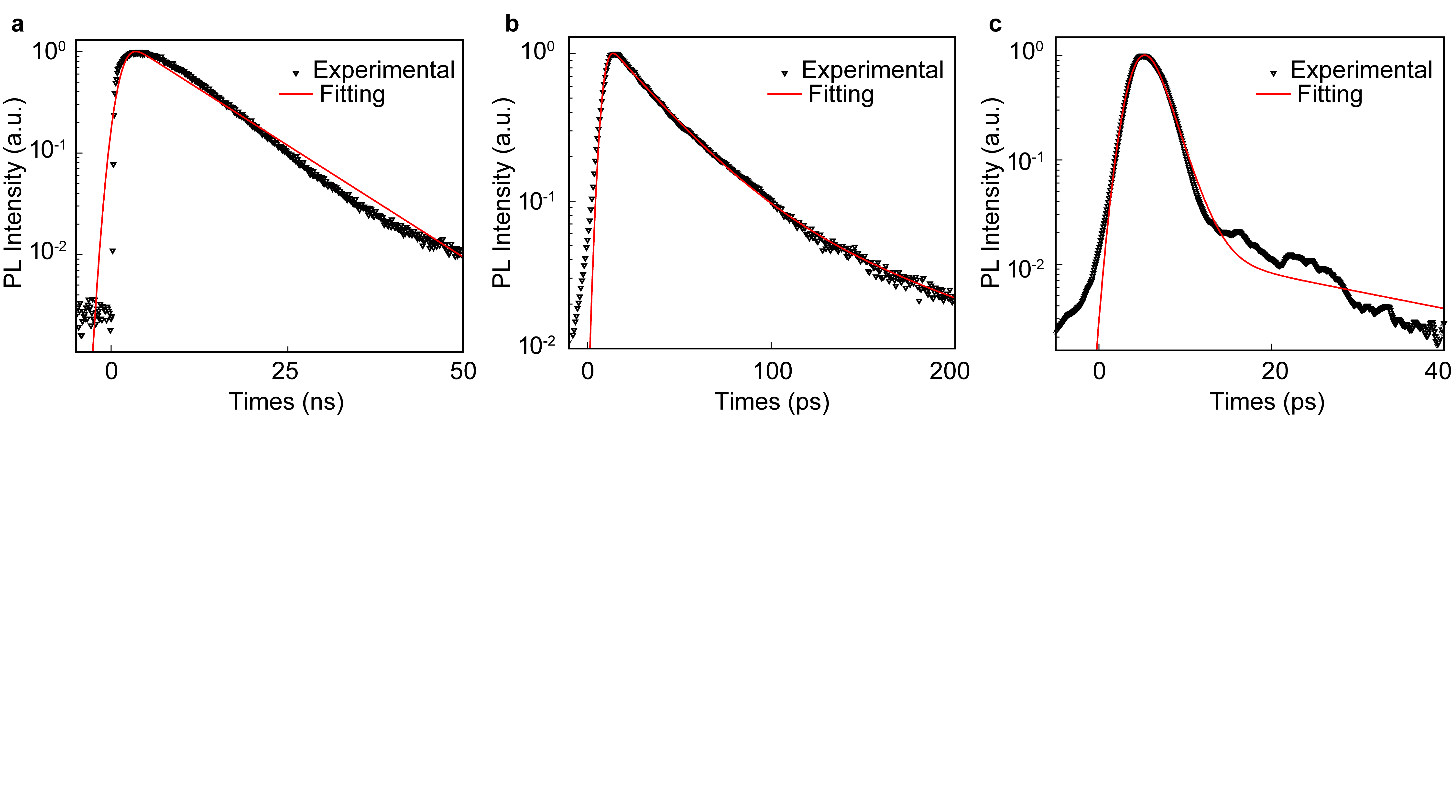


**Supplementary Figure 4. The Fitting results for the dynamic PL data in Fig 1c.** **a**, The PL of monodisperse QDs shows a single exponential decay with τ_1_= 11.9 ns. **b**, The radiation of self-cooperated excitons in shows double exponential decay with τ_1_= 31.9 ps (~90%) and τ_2_= 109.9 ps (~10%). Here, the percentage value in bracket is the ratio of amplitude for the decay channel corresponding. The fast/slow process represents the emission of cooperative/dephasing component in the exciton ensemble. **c**, The CESF signal of excitons in QDSM shows double exponential decay with τ_1_= 3.3 ps (~99%) and τ_2_= 26.7 ps (~1%). The fast decay originates from the CESF process. The CESF takes a large proportion of more than 95% within a short time interval of about 10 ps. The surplus (<3% in conservatively) is contributed by the background PL signals which is not coupled into the cavity mode. It is noted that the fluctuation of the pulse energy of pumping laser is about 5%. The surplus radiation after CESF process includes obvious fluctuation noise.


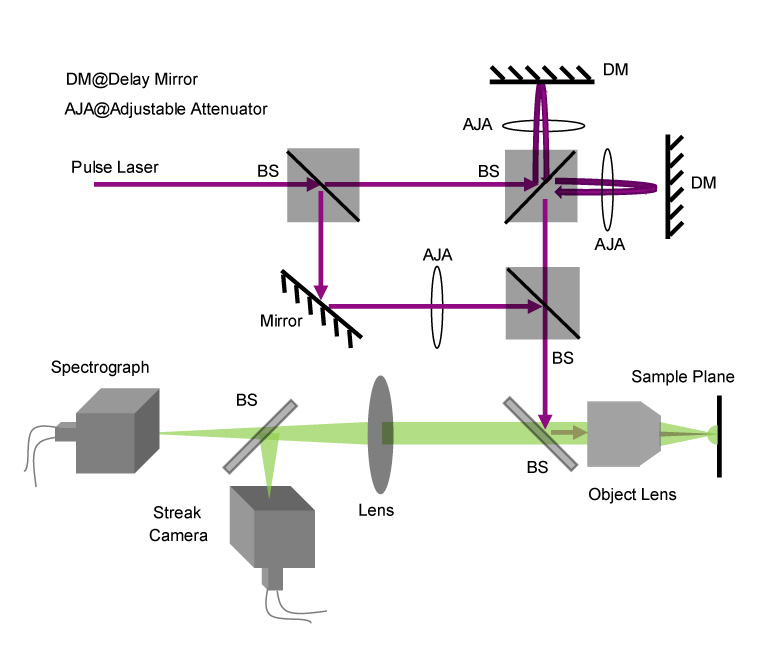


**Supplementary Figure 5. Experimental setup**. The excitation laser is divided into three pulses through two BSs, and the relative positions of the three pulses are adjusted by the translation stage. The continuously adjustable attenuator (AJA) is used to control the pumping power. Finally, the PL signal is collected by a confocal system into the time-resolved spectral system or the pure spectrometer system.

**
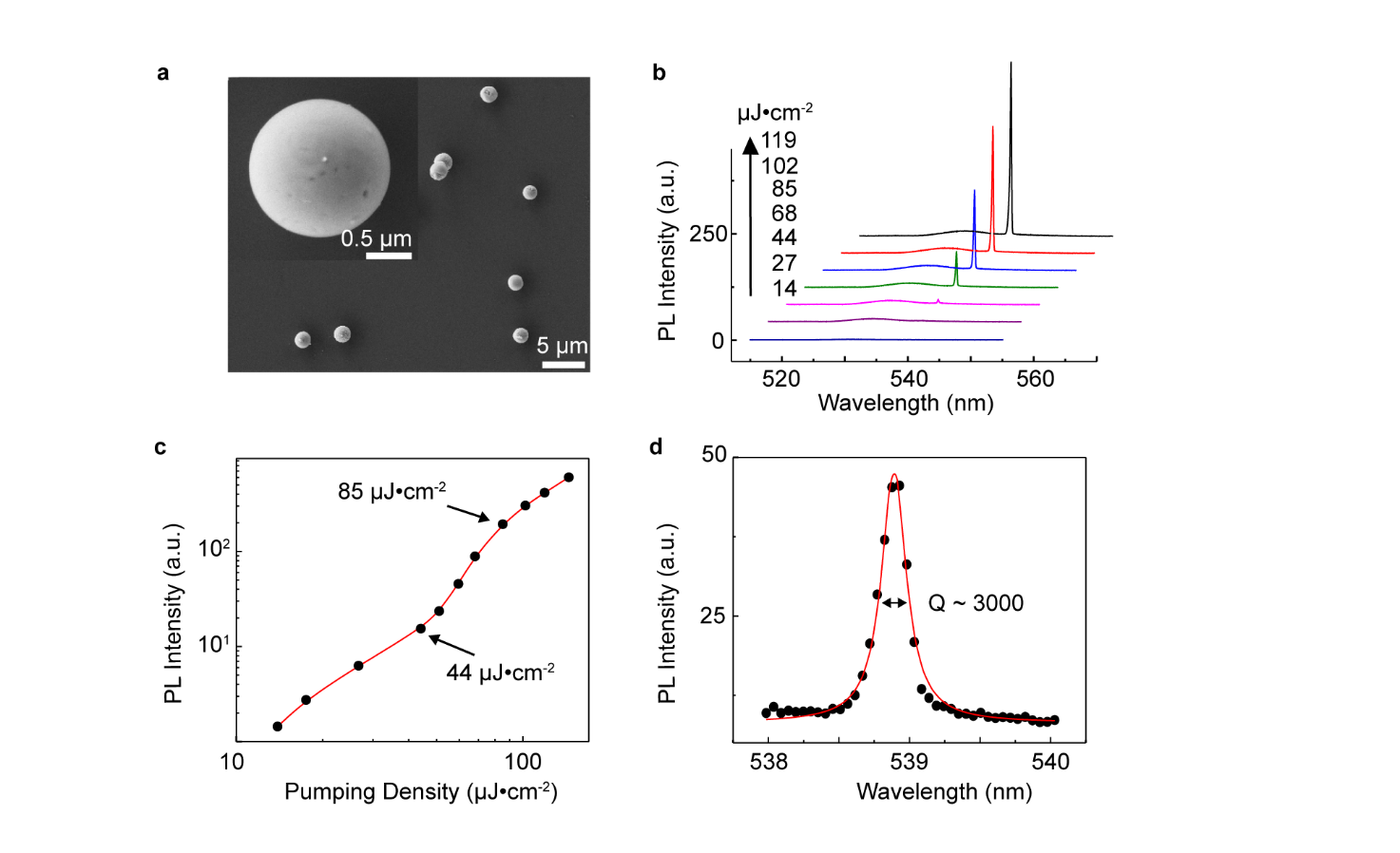
**

**Supplementary Figure 6. Sample information for the controlled experiments of classical lasing a**, SEM image of CsPbBr_3_ microspheres. Inset: the magnified image of an individual CsPbBr_3_ micro-sphere. **b**, Typical PL spectra with increasing pumping density based on a single CsPbBr_3_ microsphere. The lasing behavior is observed. **c**, The dependence of emission intensity on the pumping power. The lasing threshold is about 44 μJ∙cm^-2^. **d**, Lorentz fitting of the lasing mode. The Q factor is ~3000.

**Supplementary Note 2: Theoretical Description**

The cavity-enhanced SF behavior is treated as a Purcell effect of cooperative quantum excitons in a cavity. We use two kinds of theoretical models to simulate our experiment data. One model simplifies the excitons in QDs as the ideal dipoles in a two level system, which works like an extension of Dicke Model into a cavity case. The other model bases on the Maxwell-Bloch equations to describe the interaction of cooperative excitons and cavity light field. Meanwhile, it involves the dephasing processes by adding the corresponding fluctuation terms, and is solved numerically by Monte Carlo algorithm. We found that the two models can both fit the experiment results.

**Theoretical Model I: Dicke Model in a cavity**

Based on the *Dicke* model, we study the radiation of cooperative excitons in our samples^1,2^. Here, the excitons in QDs are simplified as the ideal dipoles in a two level system. The inhomogeneous broadening of QDs is ignored. For the free Hamiltonian of a two-level dipole we have,

(1)

where the zero energy has been chosen at the mid-point of the energy interval . The dipole moment operator $\mathop{d}\limits^{\wedge}$ can be written as an off-diagonal matrix,

$\mathop{d}\limits^{\wedge} =\left(\begin{matrix} 0 & d_{\mathrm{eg}} \\ d_{\text{ge}} & 0 \end{matrix}\right)$ (2)

All the two-level Hermitian operators can be expressed in terms of the Pauli matrices and the unit $2\times2$ matrix,

$\mathop{\sigma_{1}}\limits^{\wedge} =\left(\begin{matrix} 0 & 1 \\ 1 & 0 \end{matrix}\right)$ $\mathop{\sigma_{2}}\limits^{\wedge} =\left(\begin{matrix} 0 & -i \\ i & 0 \end{matrix}\right)$ $\mathop{\sigma_{3}}\limits^{\wedge} =\left(\begin{matrix} 1 & 0 \\ 0 & -1 \end{matrix}\right)$ $\mathop{\sigma_{0}}\limits^{\wedge} =\left(\begin{matrix} 1 & 0 \\ 0 & 1 \end{matrix}\right)$ (3)

In particular, in accordance with (1) and (2)

$\mathop{d}\limits^{\wedge} =\mathrm{Re}d\sigma^{\wedge} _{1}+\mathrm{Im}d\sigma^{\wedge} _{2}$ (4)

where $\mathrm{Re}d$ and $\mathrm{Im}d$ are the corresponding real and imaginary parts of $d_{\text{ge}}$·

Let us introduce the quasi-spin operators for the $i$th dipole

(5)

and the total quasi-spin operators of the *N*-dipole system

$\alpha=1,2,3$ (6)

which obey the usual commutation rules of angular momentum operators

$\left[\mathop{R}\limits^{\wedge} _{\alpha},\mathop{R}\limits^{\wedge} _{\beta}\right]=i\mathop{R}\limits^{\wedge} _{\gamma}$ (7)

Where$\alpha,\beta,\gamma$is any cyclic permutation of the numbers$1,2,3$. Then the energy

operator of an ensemble of $N$ free identical two-level dipoles can be expressed in the form

(8)

And its eigenvalues are equal to , where $M=\frac{1}{2}N,\frac{1}{2}N-1,,-\frac{1}{2}N$ is the eigenvalue of $\mathop{R}\limits^{\wedge} _{3}$.

The Hamiltonian for the interaction of such an$N$-dipole system with the electromagnetic field can be chosen to be in the form

(9)

where$\varepsilon^{\wedge}$is the operator of the electric field, is the electric dipole moment operator of the $i$th dipole,.

All dipoles are initially in their excited states. The radiative decay of such an ensemble is a cascade of transitions between the adjacent states with the same eigenvalue of equal to $\frac{1}{2}N(\frac{1}{2}N+1)$

$|\frac{1}{2}N,\frac{1}{2}N\rangle\to|\frac{1}{2}N,\frac{1}{2}N-1\rangle\to\cdots\to|\frac{1}{2}N,-\frac{1}{2}N\rangle$ (10)

The probability per unit time, $\gamma_{M,M-1}$for a transition$M\to M\text{-}1$, can be obtained,

$\gamma_{\text{M},\text{M}-1}=\gamma(\frac{1}{2}N+M)(\frac{1}{2}N-M+1)$ (11)

**(a) For the SC samples without cavity effect:**

$\gamma$ ***is the spontaneous radiation rate of a single dipole***. Finally, we can write the rate equations for the case of an *N* dipole ensemble ***without optical cavity***,

$\begin{matrix} \frac{\text{d}P_{\text{M}}(t)}{\text{d}t}=\gamma_{\text{M+1},\text{M}}P_{\text{M+1}}(t)-\gamma_{\text{M},\text{M-}1}P_{\text{M}}(t) \\ (M=\frac{1}{2}N,\frac{1}{2}N-1,\cdots,-\frac{1}{2}N) \end{matrix}$ (12)

Where $P_{\text{M}}(t)$ is the probability of finding the system in the state$|\frac{1}{2}N,M\rangle$ at time *t*. With the aid of equations (12) we can obtain the mean probability radiation rate,

$\overline{I}\left(t\right)=\sum_{M=-\frac{1}{2}N+1}^{N/2} \gamma_{\text{M},\text{M-}1}P_{\text{M}}\left(t\right)$ (13)

**(b) For the QDSM samples with cavity effect:**

When the cooperative dipoles are placed into an ***optical cavity***, the parameter $\gamma$ in equation (11) needs to ***enlarge by a factor N_p_***, i.e., the effective photon number coupling with dipoles, or the amplification factor of optical field density between the cases ‘with cavity’ and ‘without cavity’. And ***we need to add a new equation into the rate equations (12) to describe the dynamics of*** $N_{P}$,

$\frac{dN_{P}}{dt}=\overline{I}\left(t\right)-N_{P}/t_{C}$ (14)

Here, $t_{c}$ is the photon lifetime of optical cavity. In addition, a filling factor *f* describes the ratio between the number *N* of available hosts for cooperative dipoles and the initially excited dipole number *N*_0_ in the system.

We use the following parameters to obtain the numerical solutions, the excited dipole number by pumping in a cooperative volume $N_{0}\in\left[ 0\sim50 \right]$, the filling factor $f=3$, the spontaneous radiative rate $\gamma=0.5 \mathrm{ns}^{-1}$, the cavity photon lifetime $t_{C}=2.5 ps for the QDSM sample$ and $t_{C}=5 fs for the SC sample.$The theoretical results are plotted in **Fig. 3g,h**. The simplified model doesn’t include the setup process of cooperative state from a hot dense exciton gas. This time is typically ~1 ps in our samples, which is lower than the limitation of the detecting steam camera. No other loss of dipoles is considered except the radiation from the system.

**Theoretical Model II: Maxwell-Bloch equations of dephasing fluctuation terms**

In a cavity system, under the rotating wave and dipole approximation, the Hamiltonian of the *e-h-p* system could be written as:

in which $a$ is the annihilate operator of the cavity optical field and $\omega$ is its frequency. $N$ is the effective exciton number in the excited system. $D_{j}$ and $P_{j}$ are the inversion population difference operator and the dipole transition operator of the *j*th exciton, respectively. $G$ is the coupling strength between the cavity photons and the excitons. The cavity field could couple the excitons in the material together and makes them form a collective state. This state will induce a cooperative radiation superfluorescence. The homogeneous /inhomogeneous broadening of the excitons will induce a smaller coupling strength $G$. Above all, the Hamiltonian will be simplified under the mean-field approximation:

From Eq. (1) to Eq. (2), the role of *G* is transferred to the parameter *g* through $G\sum_{j=1}^{N} P_{j}^{+}\to g*NP^{\dagger}=g_{0}\beta*NP^{\dagger}={g_{0}*N}_{eff}$, where $g_{0}$ is the normal coupling strength for individual QD excitons, $N_{eff}$ is the effective numbers of cooperative dipoles. The dynamic equations are derived as

$$\frac{da}{dt}=-i\omega a-igNP-\Gamma_{c}a+K$$

$$\frac{dD}{dt}=-a_{sp}\left( D+1 \right)+2g\left( P^{\dagger}a-a^{\dagger}P \right)-\kappa\left( D+1 \right).$$

Here, the decay terms and the random fluctuation are added. $\Gamma_{s}$ is the decay rate of the collective dipole moment which reflects the dephasing process of excitons. $a_{sp}$ is the spontaneous radiation coefficient. The two decay terms above will correspond to two types of noise respectively, whose form are defined as

$$\left\langle K^{*}(t)K(t') \right\rangle=a_{sp}\frac{\left( D+1 \right)N}{2}\delta\left( t-t^{'} \right)$$

Besides, $\Gamma_{c}$ is the decay rate of the cavity photon. Comparing with the other noise in the system, its noise is much weak and ignored in actual numerical calculation. $\kappa$ is the non-radiative decay rate of the inversion population.

At the initial moment, excitons are excited. The initial optical field is $0$ and the macroscopic dipole moment of the material composition in the system has a very small value. The initial condition of the Eq. 3 are given as

$$E\left( t=0 \right)=0$$

$$D\left( t=0 \right)=1.$$

These stochastic differential equations Eq. 3 are solved by Monte Carlo algorithm by averaging thousands of stochastic dynamic trajectories. The CESF curves in Fig. 3g,h could be fitted best with the lifetime of the cavity photon as $1/\Gamma_{c}=1.67 ps$ and designating other parameters as follows:$\alpha_{\mathrm{sp}}=3.5\times{10}^{-4} {ps}^{-1}$, $\kappa=1/1200 {ps}^{-1}$, $g=0.036$, $\Gamma_{s}=0.017 {ps}^{-1}$ (see **Supplementary Figure 7a,b**). When the quality factor of the cavity is bad, the radiation action will become a common SF. Here by setting $1/\Gamma_{c}=0.36 ps$ and designating other parameters as $\alpha_{\mathrm{sp}}=1.5\times{10}^{-4} {ps}^{-1}$, $\kappa=1/1200 {ps}^{-1}$, $g=0.06$, $\Gamma_{s}=0.008 {ps}^{-1}$, the SF curves in **Fig. 3g,h** could be fitted (see **Supplementary Figure 7c,d**).


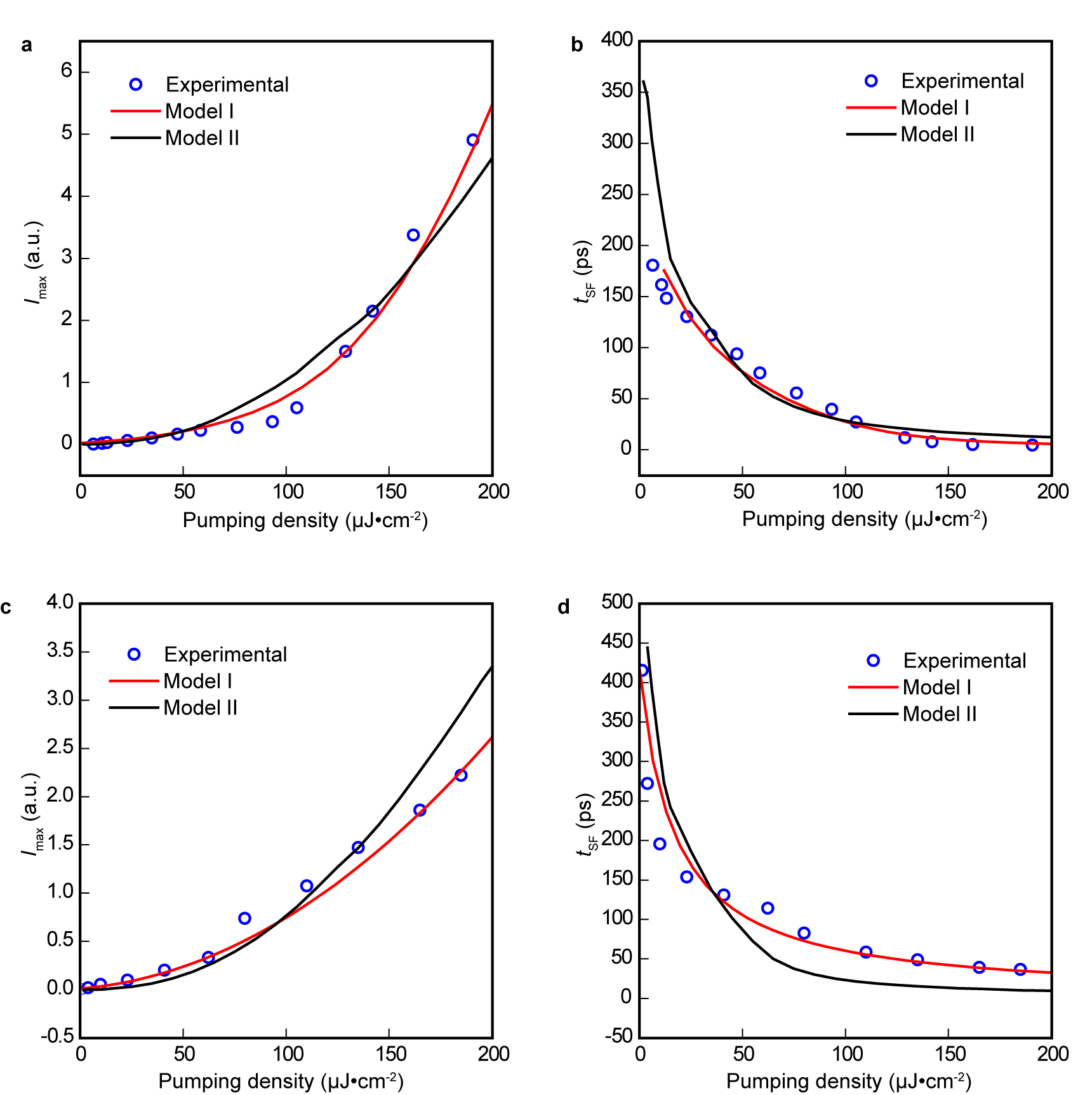


**Supplementary Figure 7.** The pumping density VS the characteristic radiation time $t_{r}$ and the peak intensity $I_{\max}$ of CESF effect (**a**, **b**) and SF effect (**c**, **d**). The theoretical results of two different models are shown. The dots are experimental data.

**Supplementary References**

1. Benedict, M. G., Ermolaev, A. M., Malyshev, V. A., Sokolov, I. V. & Trifonov, E. D. *SUPER-RADIANCE MULTIATOMIC COHERENT EMISSION*. Institute of Physics Publishing, London (1996).
2. Dicke, R. H. Coherence in spontaneous radiation processes. *Phys. Rev*. **93**, 99–110 (1954).
